# Epidemiology and Risk Factors for Orthostatic Hypotension and Its Severity in Residents Aged > 60 years: A Cross-Sectional Study

**DOI:** 10.1155/2024/9945051

**Published:** 2024-02-27

**Authors:** Mingni Yang, Ruiqiang Peng, Zetuo Wang, Miaoduan Li, Yehua Song, Jianping Niu, Yong Ji

**Affiliations:** ^1^Department of Neurology, The Second Affiliated Hospital of Xiamen Medical College, Xiamen, China; ^2^Tianjin Key Laboratory of Cerebrovascular and Neurodegenerative Diseases, Department of Neurology, Tianjin Dementia Institute, Tianjin Huanhu Hospital, Tianjin, China

## Abstract

This cross-sectional study investigated the epidemiology and risk factors associated with orthostatic hypotension (OH) and its severity in older adults residing in the Jizhou community of Tianjin and the Jimei community of Xiamen. The study, conducted from March to September 2019, involved adults aged over 60. A comprehensive questionnaire survey was administered, resulting in the enrolment of 4383 older adults. The overall prevalence of OH was found to be 11.7% (516 out of 4383). Notably, a significant gender difference was observed, with a prevalence of 10% among males (194 out of 1926) and 13.1% among females (322 out of 2457) (*P*=0.002). Among individuals with OH, 332 exhibited mild symptoms, 64 had moderate OH, 58 had severe OH cases, and 50 have very severe OH. Multivariable logistic regression analysis revealed that being female, widowed, engaging in general social activities, and a history of hypertension, migraines, heart disease, cerebrovascular disease, and mental health conditions (anxiety and depression) were independently associated with OH. Ordinal logistic regression analysis further confirmed that hypertension, migraine, and a history of general anesthesia surgery were independently associated with the severity of OH. This study highlights a relatively high prevalence of OH among older adults in the Jizhou community of Tianjin and the Jimei community of Xiamen, China. The identified risk factors, particularly social activities, and hypertension, significantly influence the severity of OH. Further examination is required to corroborate these findings and investigate potential interventions.

## 1. Introduction

Orthostatic hypotension (OH) is characterized by a sudden drop in blood pressure (BP), typically occurring within 3 minutes of transitioning from a supine or decubitus position to an upright, orthostatic one, or during prolonged periods in the upright position [1]. This phenomenon arises from the failure of compensatory mechanisms that normally buffer BP fluctuations [2]. Severe OH is often accompanied by organ hypoperfusion, syncope, transient ischemic attacks, and an inadequate supply of blood to the heart, thereby elevating the risk of cardiovascular diseases and mortality [3, 4].

The prevalence of OH tends to rise with advancing age [5]. Occurrence rates vary depending on the region and the age group under consideration, ranging from 9% to 34% among individuals aged 65 to 75 years or older in unselected community-dwelling populations [6]. Notably, there is a shortage of studies conducted among community residents in China estimating the prevalence of OH in elderly populations. Since 2008, only few small-sample studies have been conducted in different-grade hospitals [7]. This underscores the necessity for up-to-date community-based data in older populations to inform targeted interventions.

Common causes of OH encompass illnesses, autonomic dysfunction, cardiovascular impairment, endocrine disorders, and certain medications (such as antihypertensives, antiadrenergic, anticholinergics, antianginals, antiarrhythmics, and antidepressants) [2, 8]. Developing targeted interventions to reduce OH incidence requires assessing relevant risk factors in specific populations. Research findings regarding OH caused by Parkinson's disease and other neurodegenerative diseases are well-established. However, there are limited reports on OH in the general elderly population. Given the absence of epidemiological data on OH in Xiamen and Tianjin City, China, the present study seeks to investigate the occurrence of OH and its severity in the broader elderly population, emphasizing individuals lacking readily identifiable conditions causing OH. This investigation highlights the importance of identifying independent risk factors associated with OH and its severity in older adults without neurodegenerative diseases disposed to OH.

## 2. Methods

### 2.1. Study Design and Participants

This cross-sectional study enrolled individuals aged >60 years residing in the Jizhou community of Tianjin and the Jimei community of Xiamen between March and September 2019. Ethical approval for the study was obtained from the Ethics Committees of Tianjin Huanhu Hospital and the Second Affiliated Hospital of Xiamen Medical College. All participants provided written informed consent.

Inclusion criteria comprised the following: (1) permanent residency (i.e., individuals residing in Tianjin and Xiamen with registered permanent residence in these respective cities), (2) capability to cooperate in completing questionnaires and clinical examinations, and (3) voluntary participation with the signing of informed consent forms. Exclusion criteria encompassed (1) the presence of malignant arrhythmia, severe valvular heart disease, severe heart failure (New York Heart Association (NYHA) functional classes III and IV), or malignant tumors, (2) diagnoses of Lewy-body dementia, Parkinson's disease, multisystem atrophy, or other neurological conditions that elevate susceptibility to OH, (3) use of medications affecting autonomic nervous function, such as antiparkinsonian drugs (pramipexole and levodopa), anticholinergic drugs (atropine and anisodamine), tricyclic antidepressants (amitriptyline, imipramine, clomipramine, and desipramine), and *α*-blockers (doxazosin, terazosin, reserpine, and prazosin), and (4) bedridden or inability to communicate. The analysis of factors influencing the severity of OH involved selecting a population with OH from the overall study participants for in-depth analysis.

### 2.2. Procedure

This study employed a standardized questionnaire administered across multiple centers in the selected cities. A team of 40 trained and certified medical personnel and community workers conducted door-to-door questionnaire surveys and face-to-face interviews with respondents involving at least 3,000 visits, employing consistent and uniform language guidance.

The comprehensive questionnaire (Additional file 1) covered various aspects. The sociodemographic characteristics of participants included age, gender, occupation, handedness, education level, and marital status. Lifestyle factors consisted of engagement in social activities, living arrangements, and smoking and alcohol habits. The medical history section covered prior general anesthesia across the entire life span, migraines, heart conditions like coronary heart disease and arrhythmia, diabetes, hypertension, hypertension control status, cerebrovascular disease including cerebral hemorrhage, subarachnoid hemorrhage, and cerebral infarction, and psychiatric diagnoses encompassing anxiety and depression.

### 2.3. Orthostatic Hypotension Assessments

All BP measurements were obtained using the same electronic BP monitor, equipped with an appropriately sized cuff positioned at the heart level on the forearm. BP was measured after a minimum of a 10-minute rest in a seated or supine position. Participants were asked to stand, and BP was obtained at the first and third minutes during the standing period. The criteria for diagnosing OH followed international guidelines consistent with those used for clinic-based OH assessments: a sustained reduction of >20 mmHg (1 mmHg = 0.133 kPa) in systolic blood pressure (SBP) and/or >10 mmHg in diastolic blood pressure (DBP) within 3 minutes of assuming an upright position [9].

OH severity was categorized as follows: mild (infrequent OH episodes with no impact on activities of daily living), moderate (frequent OH, occurring at least once a week, with some limitations in activities of daily living), severe (frequent OH, the ability to stand for >1 minute compromised, and significant limitations in activities of daily living), and very severe (frequent OH, the ability to stand for <1 minute compromised, and syncope often occurring upon attempting to stand) [10].

### 2.4. Other Clinical Information

Hypertension was diagnosed based on SBP ≥ 140 mmHg and/or DBP ≥ 90 mmHg or the use of antihypertensive medications. BP control was classified as either good (<140/90 mmHg after antihypertensive treatment) or poor (>140/90 mmHg after antihypertensive treatment). Social activity levels were identified according to the average weekly exercise duration, categorized as none (limited physical activity per week), rare (1-2 hours weekly), few (3-4 hours weekly), general (5–7 hours weekly), and abundant (more than 7 hours weekly). Marital status was categorized as spinsterhood, married, divorced, and widowed. Sex, age, smoking status, alcohol drinking, heart conditions, diabetes, and cerebrovascular disease were self-reported.

Anxiety was assessed using the Self-Rating Anxiety Scale (SAS). The SAS had 20 items, and each item was scored, ranging from 1 (none or a little of the time) to 4 (most or all of the time). A threshold of 50 was employed to indicate that a person suffered from anxiety symptoms. Depression was gauged using the Center for Epidemiologic Studies Depression Scale (CES-D). The CES-D scale contains a total of 20 items, with the overall score ranging from 0 to 60. A score of 16 or higher is generally defined as indicating positive depression symptoms. Migraine was defined based on the International Classification of Headache Disorders, 3rd edition [11]. All participants were asked questions regarding clinical features of headache including duration, frequency, intensity, location, accompanying symptoms, and aggravation following activity.

### 2.5. Sample Size

The sample size was estimated using the epidemiological formula: *n* = 400 (1–P)/P, where P represented the estimated prevalence. Previous reports indicate an average OH prevalence of 24.3% among adults aged ≥ 50 years in China [7, 12]. Based on this calculation, a sample size exceeding 1246 was deemed necessary.

### 2.6. Statistical Analysis

Statistical analysis was conducted using SPSS 22.0 (IBM, Armonk, NY, USA). Continuous variables were described as mean ± SD or as median values with an interquartile range, depending on the distribution of the variables (as assessed by the Kolmogorov–Smirnov test). Continuous data were compared using Student's *t*-test and the Mann–Whitney *U* test. Categorical variables were presented as *n* (%) and compared using the chi-squared test or Fisher exact test. Variables with a *P* value <0.05 in univariate logistic regression analysis were included in multivariate logistic regression analysis. Multivariate logistic regression models were used to explore the associations between factors and OH. An ordinal logistic regression model was employed to analyze factors influencing the severity of OH. A two-tailed *P* value <0.05 was considered statistically significant.

## 3. Results

**Figure 1:**
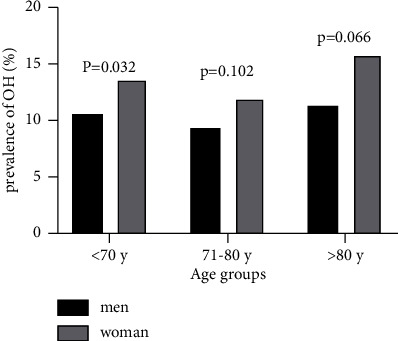
The influence of sex and age stratification in OH.

Finally, a total of 4,699 individuals aged over 60 years were initially selected for this study. Questionnaires were distributed to all 4,699 participants, and 4,599 valid responses were received. Of these, 20 individuals had an unknown medical history, 40 did not cooperate, 16 had communication disorders affecting speech, and 24 had hearing impairments. Consequently, the final study cohort comprised 4,383 participants (1,926 males and 2,457 females) with an average age of 72.1 ± 6.1 years. The overall prevalence of OH was 11.7% (516 out of 4,383), with rates of 10.0% (194 out of 1,926) in males and 13.1% (322 out of 2,457) in females (*P*=0.002). Among participants, the prevalence of OH was 13.6% (258 out of 1,890) for those aged 60–70 years, 12.4% (190 out of 1,527) for those aged 70–80 years, and 15.1% (68 out of 450) for those over 80 years. Notably, the prevalence of OH was higher in females than in males among those under 70 years of age (*P*=0.032), while there were no significant differences in OH prevalence between males and females aged 70–80 years and over 80 years (Figure 1).

Univariate analyses revealed potential risk factors associated with OH, including sex, marital status, engagement in social activities, hypertension, BP control, migraine, heart disease, cerebrovascular disease, history of general anesthesia, and mental health conditions (all *P* < 0.05) (Table 1). Multivariate logistic regression analysis identified female gender (compared to male, OR = 1.46, 95% CI: 1.12–1.89, *P*=0.005), widowhood (compared to being single, OR = 2.84, 95% CI: 1.28–6.32, *P*=0.010), participation in general social activities (compared to none, OR = 1.49, 95% CI: 1.07–2.07, *P*=0.017), hypertension (OR = 1.44, 95% CI: 1.36–2.42, *P*=0.024), cerebrovascular disease (OR = 1.43, 95% CI: 1.21–1.56, *P*=0.042), migraine (OR = 1.55, 95% CI: 1.39–1.77, *P*=0.001), heart disease (OR = 1.58, 95% CI: 1.46–1.73, *P* < 0.001), and mental health conditions (anxiety and depression) (OR = 1.36, 95% CI: 1.22–2.28, *P* < 0.001) as independent factors associated with OH (Table 2).

Regarding the severity of OH, out of the total 516 OH patients, 12 were excluded due to their inability to accurately describe OH symptoms, resulting in 504 patients being included in the final analysis. The cohort included 332 patients with mild OH, 64 with moderate OH, 58 with severe OH, and 50 with very severe OH. Significant differences were observed in terms of sex (*P*=0.007), engagement in social activities (*P*=0.030), hypertension (*P*=0.009), migraine (*P*=0.002), smoking (*P* < 0.001), alcohol consumption (*P* < 0.001), and history of general anesthesia surgery (*P*=0.004) among patients with different OH severities (Table 3). Ordinal logistic regression analysis further confirmed that hypertension (OR = 0.282, 95% CI: 0.152–0.533, *P*=0.040), migraine (OR = 0.386, 95% CI: 0.215–0.692, *P*=0.001), and a history of general anesthesia surgery (OR = 0.583, 95% CI: 0.373–0.909, *P*=0.017) were independently associated with the severity of OH (Table 4).

## 4. Discussion

This study reveals a prevalence rate of 11.7% among elderly residents in the Jizhou community of Tianjin and the Jimei community of Xiamen, China. Recent studies have indicated that the incidence of OH in patients with Parkinson's disease is as high as 30% to 50% [13, 14]. After excluding individuals with neurodegenerative diseases, an increased risk of OH persists among elderly individuals. Factors independently correlated with OH include female gender, widowhood, participation in general social activities, hypertension, migraine, cerebrovascular disease, heart disease, and mental health conditions (depression and anxiety). These findings provide crucial insights for developing effective interventions to prevent OH in elderly individuals.

Previous research has indicated a positive correlation between age and OH prevalence in older adults [15, 16]. However, a study by Hiitola et al. [17] in a community of older adults found no significant correlation between age and OH prevalence. Similarly, the present study demonstrates that OH prevalence in individuals over 60 years of age is not significantly associated with age.

Méndez et al. [18] reported that OH prevalence is similar between women and men aged 55–74, with the proportion of women affected by OH being lower than that of men after the age of 75. In contrast, Romero-Ortuno et al. [18] noted that OH prevalence is higher in females than in males among young and middle-aged individuals but does not differ concerning gender in older adults. In our study, OH prevalence in women under 70 was higher than in men, while no gender difference was observed in prevalence rates among individuals over 70 years old. Nevertheless, it is worth noting that the overall severity of OH in elderly women was greater than that in men. OH, as a common manifestation of autonomic nervous dysfunction, tends to affect women more frequently, possibly due to factors such as female hormones, social factors, anxiety, depression, and mental stress [19, 20].

While previous studies have consistently highlighted diabetes as a common and significant risk factor for OH [21], diabetes did not exhibit a statistically significant association with an increased prevalence of OH in our study. This discrepancy may be attributed to specific characteristics of the study population, including dietary habits, robust medical services in coastal regions, and awareness of active blood sugar control. Notably, our study underscores the importance of hypertension and cerebrovascular disease as risk factors for OH, aligning with prior research [22, 23]. However, Eschlböck et al. [24] reported a less precise relationship between OH and cerebrovascular diseases. Clinical observations commonly encounter patients with essential hypertension who also exhibit OH. Our study reinforces the positive correlation between OH severity and hypertension, with elevated OH severity observed in hypertensive patients. Consequently, it is imperative to monitor and manage BP in older adults experiencing postural hypotension. Chisholm et al. [6] noted that OH is not only linked to hypertension but is also influenced by antihypertensive treatments. While regular medication for BP control can reduce OH prevalence [17], numerous extensive studies have indicated that antihypertensive medications may elevate the risk of adverse reactions associated with OH [25–27]. The long-term use of antihypertensive agents in the elderly remains a topic of debate, as it may exacerbate orthostatic intolerance and potentially cause persistent falls. However, Eigenbrodt et al. [28] discovered that OH is not closely correlated with antihypertensive management. The relationship between OH and antihypertensive drugs remains inconclusive and warrants further investigation.

The findings of this study establish a significant link between the incidence and severity of OH and migraines. Vascular headaches often coincide with neurovascular dysfunction and irregularities in the autonomic nervous system. The pathological mechanisms underlying migraines involve alterations in trigeminal nerve function, autonomic nervous system activity, and thalamic sensitivities [29]. In addition, this study identifies general anesthesia as a notable risk factor for more severe OH. This phenomenon may be attributed to changes in intrathoracic pressure and lung volume induced by mechanical ventilation during general anesthesia, subsequently affecting cardiac autonomic nerve activity through reflex actions triggered by cardiopulmonary receptors or baroreceptors in major blood vessels [30]. It is plausible that narcotic drug suppression and surgical stimulation may also impact autonomic nerve function.

A noteworthy finding of this study is that widowed individuals are more likely to develop OH. Currently, there are no direct studies on the relationship between marital status and OH. We hypothesize that widowhood and OH may be correlated due to overlap in several factors. First, individuals living alone may lack someone to assist them in monitoring their health status, including blood pressure. This absence of monitoring could lead to the progression of chronic diseases such as hypertension, potentially damaging the autonomic nervous system [31]. Second, individuals with deceased spouses may encounter challenges in managing their medications, leading to adverse drug reactions or improper use, which could trigger or exacerbate OH. Third, widowhood can lead to social isolation, elevated stress, irregular eating and sleeping patterns, and depression. These factors can influence the function of the autonomic nervous system, a key regulator of blood pressure.

The prevalence of OH and intolerance to an upright posture has been reported to increase following prolonged bed rest [32]. Social support and positive social interactions can induce the release of oxytocin, enhancing the activity of the sympathetic and parasympathetic nervous systems [33, 34]. In addition, central oxytocin has demonstrated its ability to reduce stress-induced corticosterone release and anxiety behavior [35].

Social activities often involve physical engagement, such as walking, sports, or outdoor activities, contributing to cardiovascular health and blood pressure regulation. Sympathetic activation and involuntary muscle contraction can enhance the hemodynamic response of OH [36], providing a potential explanation for why social activities serve as a protective factor in our study. However, our findings do not align with a meta-analysis that did not establish a connection between OH and walking speed or physical activity. Evidence suggests that acute, intense exercise may transiently improve orthostatic tolerance in individuals with chronic autonomic dysfunction, although high-intensity training has not ameliorated orthostatic intolerance [32]. Conversely, Xu et al. [37] reported improved OH following aerobic exercise training in elderly individuals. A more extended follow-up in our study is required to discern variations in the impact of different social activities on elderly individuals with OH.

This study contributes to the identification of counter preventive measures. First, it underscores the importance of appropriate aerobic training for individuals aged 60 years and older, especially those who have experienced the loss of a spouse or have a history of mental illness. Activities such as yoga and Taijiquan can be employed to regulate the hypothalamic-pituitary-adrenal axis and the sympathetic nervous system, thus promoting both physical and psychological well-being [38]. Second, community clinics should consider implementing regular BP monitoring and ensuring rational medication management for elderly individuals, particularly those at higher risk. Last, patient education should emphasize the importance of monitoring blood pressure in orthostatic positions.

However, it is essential to acknowledge several limitations of this study. Despite the substantial sample size, the participants were drawn exclusively from two communities in China, and their characteristics may not fully represent the diverse population of the entire nation. Furthermore, due to the cross-sectional design of the study, it cannot establish cause-and-effect relationships. In addition, older adults often take a wide array of medications, making it impractical to account for each individual drug's potential impact.

In conclusion, this study highlights the elevated risk of OH among older adults residing in the Jizhou community of Tianjin and the Jimei community of Xiamen, China. Factors such as being female, having lost a spouse, engaging in general social activities, having hypertension, experiencing migraines, being diagnosed with heart disease, and having mental health conditions may contribute to an increased susceptibility to OH.

We advocate for routine screening of OH among all elderly individuals in a given community, encompassing both healthy individuals and those with intermittent and continuous BP monitoring. For elderly individuals diagnosed with OH, a comprehensive assessment focusing on mental health, psychiatric disorders, and social relationships is recommended to identify potentially modifiable risk factors. However, a further study is needed to corroborate these findings and explore potential interventions.

## Figures and Tables

**Table 1:** Characteristics of the study population.

		With OH (*n* = 516)	Without OH (*n* = 3867)	*P* value
Sex, *n* (%)	Male	194 (37.6)	1732 (44.8)	0.002
	Female	322 (62.4)	2135 (55.2)	

Age (years), *n* (%)	<70	258 (50.0)	1890 (48.9)	0.344
	70–80	190 (36.8)	1527 (39.5)	
	>80	68 (13.2)	450 (11.6)	

Marital status, *n* (%)	Spinsterhood	25 (4.8)	53 (1.4)	<0.001
	Married	391 (75.8)	2879 (74.5)	
	divorced	7 (1.4)	54 (1.4)	
	Widowed	93 (22.518)	881 (22.8)	

Social activities, *n* (%)	None	92 (17.8)	518 (13.4)	<0.001
	Rare	64 (12.4)	413 (10.7)	
	Few	136 (26.4)	813 (21.0)	
	General	165 (32.0)	1525 (39.4)	
	Abundant	59 (11.4)	598 (15.5)	

Hypertension, *n* (%)	Yes	267 (51.7)	1856 (47.9)	0.039

Hypertension control, *n* (%)	Good	157 (30.4)	1200 (45.4)	<0.001
	Bad	70 (13.6)	305 (14.5)	

Cerebrovascular disease, *n* (%)	Yes	115 (22.3)	632 (16.3)	0.001

Migraines, *n* (%)	Yes	47 (9.1)	207 (5.4)	0.001

Heart disease, *n* (%)	Yes	118 (22.9)	613 (15.9)	<0.001

Diabetes, *n* (%)	Yes	76 (14.7)	548 (14.2)	0.734

Smoking, *n* (%)	Yes	141 (27.3)	1029 (26.6)	0.730

Drinking, *n* (%)	Yes	124 (24.0)	900 (23.3)	0.703

General anesthesia surgery, *n* (%)	Yes	104 (20.2)	495 (12.8)	<0.001

Mental disease (anxiety or depression), *n* (%)	Yes	53 (10.2)	264 (6.82)	0.005

**Table 2:** Multivariable logistic regression analysis for OH.

		OR (95%CI)	*P* value
Sex	Male	Ref	
	Female	1.455 (1.121, 1.888)	0.005

Age (years)	<70	Ref	0.273
	70–80	0.976 (0.796, 1.197)	0.817
	>80	1.266 (0.908, 1.765)	0.164

Marital status	Spinsterhood	Ref	0.37
	Married	0.265 (0.036, 1.973)	0.195
	Divorced	1.087 (0.856, 1.381)	0.494
	Widowed	2.842 (1.279, 6.317)	0.010

Social activities	None	Ref	0.174
	Rare	1.158 (0.778, 1.723)	0.470
	Few	1.313 (0.897, 1.922)	0.161
	General	1.492 (1.074, 2.072)	0.017
	Abundant	1.313 (0.963, 1.791)	0.085

Hypertension	Yes	1.440 (1.357, 2.419)	0.024
	No	Ref	0

Hypertension control	Good	0.541 (0.146, 2.001)	0.357
	Bad	Ref	0

Cerebrovascular disease	Yes	1.425 (1.213, 1.556)	0.042
	No	Ref	0

Migraines	Yes	1.548 (1.391, 1.769)	0.001
	No	Ref	0

Heart disease	Yes	1.583 (1.464, 1.732)	<0.001
	No	Ref	0

General anesthesia surgery	Yes	0.865 (0.681, 1.099)	0.236
	No	Ref	0

Mental disease (anxiety or depression)	Yes	1.361 (1.223, 2.283)	<0.001
	No	Ref	0

OR: odds ratio; CI: confidence interval.

**Table 3:** Characteristics of the study population with different OH severities.

		Mild (*n* = 332)	Moderate (*n* = 64)	Severe (*n* = 58)	Very severe (*n* = 50)	*P* value
Sex, *n* (%)	Male	112 (0.34)	18 (0.28)	31 (0.53)	23 (0.46)	0.007
	Female	220 (0.66)	46 (0.72)	27 (0.47)	27 (0.54)	

Age (years), *n* (%)	<70	164 (0.49)	29 (0.45)	36 (0.62)	22 (0.44)	0.150
	70–80	133 (0.40)	24 (0.38)	15 (0.26)	18 (0.36)	
	>80	35 (0.11)	11 (0.17)	7 (0.12)	10 (0.2)	

Marital status, *n* (%)	Spinsterhood	8 (0.02)	2 (0.03)	2 (0.03)	1 (0.02)	0.817
	Married	253 (0.76)	49 (0.77)	45 (0.78)	43 (0.86)	
	Divorced	5 (0.02)	2 (0.03)	0 (0.00)	1 (0.02)	
	Widowed	66 (0.20)	11 (0.17)	11 (0.19)	5 (0.10)	

Social activities, *n* (%)	None	59 (0.18)	4 (0.06)	12 (0.21)	15 (0.30)	0.030
	Rare	48 (0.14)	7 (0.11)	3 (0.05)	5 (0.10)	
	Few	84 (0.25)	26 (0.41)	12 (0.21)	11 (0.22)	
	General	104 (0.31)	18 (0.28)	22 (0.38)	16 (0.32)	
	Abundant	37 (0.11)	9 (0.14)	9 (0.16)	3 (0.06)	

Hypertension, *n* (%)	Yes	176 (0.53)	25 (0.39)	23 (0.40)	23 (0.46)	0.009

Cerebrovascular disease, *n* (%)	Yes	67 (0.20)	17 (0.26)	15 (0.26)	16 (0.32)	0.649

Migraines, *n* (%)	Yes	23 (0.07)	4 (0.06)	9 (0.16)	11 (0.22)	0.002

Heart disease, *n* (%)	Yes	68 (0.20)	16 (0.25)	17 (0.29)	17 (0.34)	0.115

Diabetes, *n* (%)	Yes	44 (0.13)	12 (0.19)	11 (0.18)	9 (0.18)	0.379

Smoking, *n* (%)	Yes	74 (0.22)	11 (0.17)	29 (0.50)	18 (0.36)	<0.001

Drinking, *n* (%)	Yes	65 (0.20)	12 (0.19)	26 (0.45)	17 (0.34)	<0.001

General anesthesia surgery, *n* (%)	Yes	64 (0.19)	7 (0.11)	13 (0.22)	19 (0.38)	0.004

Mental disease (anxiety or depression), *n* (%)	Yes	26 (0.08)	8 (0.12)	10 (0.17)	9 (0.18)	0.392

**Table 4:** Ordinal logistic regression analysis of OH severity.

	B	SE	Wald	*P* value	OR	95% confidence interval	
						Lower bound	Upper bound
Sex							
Male	−0.523	0.196	7.479	0.06	0.846	0.552	1.629
Female	Ref				Ref		
Social activities							
None	0.089	0.35	0.065	0.798	1.09	0.551	2.168
Rare	−0.511	0.402	1.614	0.204	0.599	0.272	1.319
Few	0.068	0.328	0.042	0.837	1.070	0.562	2.033
General	−0.033	0.318	0.01	0.918	0.967	0.518	1.805
Abundant	Ref				Ref		
Hypertension							
No	−1.263	0.298	4.372	0.04	0.282	0.152	0.533
Yes	Ref				Ref		
Migraines							
No	−0.951	0.298	10.218	0.001	0.386	0.215	0.692
Yes	Ref				Ref		
General anesthesia surgery							
No	−0.54	0.227	5.655	0.017	0.583	0.373	0.909
Yes	Ref				Ref		
Smoking							
No	−0.522	0.297	3.099	0.078	0.701	0.332	1.061
Yes	Ref				Ref		
Drinking							
No	−0.5	0.285	3.09	0.079	0.607	0.347	1.059
Yes	Ref				Ref		

*Note.* B: beta; SE: standard error; OR: odds ratio.

## Data Availability

The data supporting the findings of this study are available upon request from the corresponding author. Due to privacy and ethical considerations, the data are not publicly accessible.

## Supplementary Materials

Supplementary Materials Additional file 1: orthostatic hypotension in residents aged > 60 years questionnaire in the Jizhou community of Tianjin and the Jimei community of Xiamen.
