# Supplementary material for: Epidemiology and Risk Factors for Orthostatic Hypotension and Its Severity in Residents Aged > 60 years: A Cross-Sectional Study
Source: Int J Hypertens. 2024 Feb 27;2024:9945051. doi: 10.1155/2024/9945051 (PMC10914424; doi:10.1155/2024/9945051)
Supplement: Supplementary Materials — Additional file 1: orthostatic hypotension in residents aged > 60 years questionnaire in the Jizhou community of Tianjin and the Jimei community of Xiamen. [file 9945051.f1.docx]

**orthostatic hypotension in residents aged > 60 years** **Questionnaire in the Jizhou community of Tianjin and the Jimei community of Xiamen**

1. Sex：□Male □Female
2. Age：（ ）years
3. Left/right-handed：□Left □Right
4. Degree of education ：（ ）years
5. Occupation：（ ）
6. Marital status：□Spinsterhood □Married □Divorced □Widowed
7. Social activities： □None: limited physical activity per week

□Rare: 1-2 hours per week

□Few: 3-4 hours per week

□General: 5-7 hours per week

□Abundant: more than 7 hours per week

1. living situation：□offspring □spouse □Living alone □other
2. vital signs：blood pressure ___mmHg heart rate___ beats / min Height：__ m weight：__ kg
3. Family history： □None

□dementia

□epilepsy

□Cerebrovascular disease

□Heart disease

□Hypertension

□diabetes

□other：____

1. Personal history： □None

□obesity

□Smoking

□Alcohol

□Trauma psychic

□general anesthesia surgery

□Metal pesticide exposure

□Mental disease（anxiety and depression）

□Traumatic brain injury

□History of carbon monoxide poisoning

□other：____

12. Previous history：□None

□cerebral infarction

□cerebral hemorrhage

□subarachnoid haemorrhage

□diabetes

□Heart disease □arrhythmia: _____

□coronary heart disease

□heart failure:1. NYHA I

2. NYHA Ⅱ

3.NYHAⅢ

4.NYHAⅣ

□epilepsy

□Hypertension □yes □no

antihypertensive drugs：______

take medicine regularly：□yes □no

Blood pressure control：□good □poor

□migraines

□alzheimer disease

□Lewy-body dementia

□Parkinson’s disease

□multisystem atrophy

□tumours:_____

□others:_______

13. medications affecting autonomic

□antiparkinsonian drugs: pramipexole, levodopa

□anticholinergic drugs: atropine, anisodamine

□tricyclic antidepressants: amitriptyline, imipramine, clomipramine, desipramine

□α-blockers: doxazosin，terazosin, reserpine and prazosin

□other

14. Orthostatic hypotension：□ yes □no

blood pressure(supine) / mmhg

blood pressure(stand 1min) / mmhg

blood pressure(stand 3min) / mmhg

15. severity of Orthostatic hypotension（ ）

| 1 | Orthostatic hypotension symptoms are rare and do not limit daily activities. |
| --- | --- |
| 2 | Frequent orthostatic hypotension symptoms, occur at least once a week, some limitation in daily activities. |
| 3 | Orthostatic hypotension symptoms occur in most cases, able to stand for more than 1 minute in most cases, most daily activities are limited. |
| 4 | Orthostatic hypotension symptoms occur in most cases, able to stand for less than 1 minute in most cases. Fainting often occurs if the patient tries to stand. |

**Evaluation criterion**

**Personal history**

**Mental disease（anxiety and depression）**

**anxiety :Zung Self-Rating Anxiety Scale (SAS)**

For each item below, please place a check mark in the column which best describes how often you felt or behaved this way during the past several days.

|  | **During the past week**: | ***Rarely*** or none of the time (less than 1 day) | ***Some*** or a *little* of the time (1-2 days) | ***Occasionally*** or a  moderate amount of  time (3-4 days) | ***Most*** or all of the  time (5-7 days) |
| --- | --- | --- | --- | --- | --- |
| 1. | I was bothered by things that usually don't bother me. |  |  |  |  |
| 2. | I did not feel like eating; my appetite was poor. |  |  |  |  |
| 3. | I felt that I could not shake off the blues even with help from my family or  friends. |  |  |  |  |
| 4. | I felt I was just as good as other people. |  |  |  |  |
| 5. | I had trouble keeping my mind on what I was doing. |  |  |  |  |
| 6. | I felt depressed. |  |  |  |  |
| 7. | I felt that everything I did was an effort. |  |  |  |  |
| 8. | I felt hopeful about the future. |  |  |  |  |
| 9. | I thought my life had been a failure. |  |  |  |  |
| 10. | I felt fearful. |  |  |  |  |
| 11. | My sleep was restless. |  |  |  |  |
| 12. | I was happy. |  |  |  |  |
| 13. | I talked less than usual. |  |  |  |  |
| 14. | I felt lonely. |  |  |  |  |
| 15. | People were unfriendly. |  |  |  |  |
| 16. | I enjoyed life. |  |  |  |  |
| 17. | I had crying spells. |  |  |  |  |
| 18. | I felt sad. |  |  |  |  |
| 19. | I felt that people disliked me. |  |  |  |  |
| 20. | I could not get going. |  |  |  |  |

**Depression Screening**

**Center for Epidemiologic Studies Depression (CES-D)**

**Scale items:**

Below is a list of some ways you may have felt or behaved. Please indicate how often you have felt this way during the last week by checking the appropriate space. Please only provide one answer to each question.

|  | **During the past week**: | ***Rarely*** or none of the time (less than 1 day) | ***Some*** or a *little* of the time (1-2 days) | ***Occasionally*** or a  moderate amount of  time (3-4 days) | ***Most*** or all of the  time (5-7 days) |
| --- | --- | --- | --- | --- | --- |
| 1. | I was bothered by things that usually don't bother me. |  |  |  |  |
| 2. | I did not feel like eating; my appetite was poor. |  |  |  |  |
| 3. | I felt that I could not shake off the blues even with help from my family or  friends. |  |  |  |  |
| 4. | I felt I was just as good as other people. |  |  |  |  |
| 5. | I had trouble keeping my mind on what I was doing. |  |  |  |  |
| 6. | I felt depressed. |  |  |  |  |
| 7. | I felt that everything I did was an effort. |  |  |  |  |
| 8. | I felt hopeful about the future. |  |  |  |  |
| 9. | I thought my life had been a failure. |  |  |  |  |
| 10. | I felt fearful. |  |  |  |  |
| 11. | My sleep was restless. |  |  |  |  |
| 12. | I was happy. |  |  |  |  |
| 13. | I talked less than usual. |  |  |  |  |
| 14. | I felt lonely. |  |  |  |  |
| 15. | People were unfriendly. |  |  |  |  |
| 16. | I enjoyed life. |  |  |  |  |
| 17. | I had crying spells. |  |  |  |  |
| 18. | I felt sad. |  |  |  |  |
| 19. | I felt that people disliked me. |  |  |  |  |
| 20. | I could not get going. |  |  |  |  |

| Scoring: | Rarely  (Less than 1 day) | Some  (1-2 days) | Occasionally  (3-4 days) | Most  (5-7 days) |
| --- | --- | --- | --- | --- |
| Questions 4, 8, 12, and 16 | 3 | 2 | 1 | 0 |
| All other questions | 0 | 1 | 2 | 3 |

The score is the sum of the 20 questions. Possible range is 0-60. If more than four questions are missing answers, do not score the CES-D questionnaire. A score of 16 points or more is considered depressed.

**previous history**

**cerebrovascular disease**

Subjects with a history of cerebral infarction and cerebral hemorrhage are requested to provide imaging data as far as possible.

**Heart failure**

Cardiac function is based on the following diagnostic criteria

Class I: Patients with heart failure who have no limitations of physical activity. They do not experience any symptoms even during strenuous exercise.

Class II: Patients with heart failure who have slight limitations of physical activity. They are comfortable at rest or with mild exertion but may experience symptoms (such as shortness of breath or fatigue) with moderate or greater exertion.

Class III: Patients with heart failure who have marked limitations of physical activity. They are comfortable at rest but experience symptoms with less than ordinary physical activity. Even simple tasks can lead to symptoms.

Class IV: Patients with heart failure who are unable to carry out any physical activity without experiencing symptoms. Symptoms may be present even at rest, and any physical activity worsens them

**Hypertension**

SBP ≥140 mmHg and/or DBP ≥90 mmHg or the use of antihypertensive medications

**Blood pressure control**

good:<140/90 mmHg following antihypertensive treatment

poor :>140/90 mmHg following antihypertensive treatment

**Migraines**

ICHD-3: International Classification of Headache Disorders-3

| Question | Answers |  |  |  |  |
| --- | --- | --- | --- | --- | --- |
| I have experienced headache attacks lasting 4-72 hours (untreated or unsuccessfully treated) | yes | no |  |  |  |
| How many times have you experienced the attacks? | More than 5 attacks | Less than 5 attacks | I have not experienced any attacks |  |  |
| Headache is characterized by (you can choose multiple answers) | unilateral location | pulsating quality | moderate or severe pain intensity | aggravation by or causing avoidance of routine physical activity (eg, walking or climbing stairs) | None of the above |
| During headache attacks, I have experienced | Nausea and/or vomiting | Photophobia and phonophobia | None of the above |  |  |
